# Poisoning by Chloroform

**Published:** 1859-08

**Authors:** 


### POISONING BY CHLOROFORM.

Letter from Dr. W. P. Bain to the Editor of the Lancet.

Sir:—The effects of chloroform by inhalation are well known, but those produced by swallowing it in large quantities are not so familiar to the profession; the following case may therefore prove interesting:

I was called at half past seven A. M., on the 27th of October, to visit Mrs. D———, who was stated to have been found insensible. I saw her immediately. She was in bed, lying on her right side, her head resting on her breast, the countenance of a death-like paleness, the mouth open, the eyelids half closed, the eyes turned up, the pupils very slightly contracted, and affected by light in the smallest perceptible degree; pulse 84, moderately full; breathing abdominal, without stertor; the surface of the body and extremities warm; total insensibility of every part. Some thin, yellowish looking fluid had apparently issued from her mouth, and which had a faint odor.

I was at a loss to say what was the precise cause of these symptoms, till, suspecting poison, I looked round the room and found a three-ounce bottle marked "chloroform," with about half a drachm remaining. It was evident this agent had been employed.

I immediately used the stomach-pump, injecting about a pint of warm water, which I had the satisfaction of finding was rejected, tinged with a considerable quantity of bile. Sinapisms were applied to the neck, chest, and feet. She continued perfectly insensible and motionless for many hours, with the exception that, at intervals of about a quarter of an hour, a convulsive motion would be seen in the throat, the mouth drawn down, and vomiting would take place with great difficulty; respiration being stopped for about thirty seconds, the lips and face becoming turgid and blue, till at length a prolonged and stertorous inspiration would be taken, and the breathing by degrees resume its former regularity. The intervals of these attacks became longer as time went on, till about 1 P. M., when the eyelids and hands moved slightly on the irritation of the eye, and the pulse had risen to 100.

On seeing her at 3 P. M., she was evidently more sensible; pulse 100; the forearm and fingers livid-looking, and inclined to be cold; feet warm; had been very sick, and thrown up a great quantity of bile. Six P. M.—Pulse 120; able to answer questions; great tenderness at epigastrium; fæces and urine had passed voluntarily, the latter in large quantity; the odor of chloroform very strong in the breath. Half past 10 P. M.—Pulse 160; skin warm; no headache; very thirsty, and still inclined to sleep. Says that in consequence of some domestic unpleasantness, she rose at 3 A. M., and poured out a wine-glassful of chloroform (which she had obtained from the apartment of a lodger) into a tumbler, added an equal quantity of water, and drank it off. She says that she put out the candle and laid down again in bed.

The narcotic effects of the poison having now passed off, my efforts were directed to mitigate, if possible, the inflammation of the stomach now set up by its corrosive action, but with very little effect; for in spite of the effervescing and iced drinks, counter-irritation, anodynes, &c., she suffered for a week under all the symptoms of acute gastritis, and died on the eighth day after taking the poison. I may state that thirty-six hours after taking it, the odor was still to be perceived in her breath, showing that the quantity must have been considerable. The attendant's evidence also showed that the quantity she mentioned having taken was the same they had noticed the day before as remaining in the bottle.

*Post Mortem Examination forty hours after Death.*—The body was much emaciated; skin of a yellowish green hue, except on the depending parts, which were much congested; the fingers and nails very dark; abdomen tympanitic; muscles of the natural color; the stomach and intestines distended with gas, but in natural position; liver pale and finely mottled. The surface of the stomach much congested, and it was much contracted about the middle portion, forming two distinct pouches; when opened, an escape of gas, possessing a very ammoniacal smell, took place; the internal coats were highly inflamed; the rugæ of the depending portion larger than usual, and very pulpy; the mucous and muscular coats for about a circle of two inches in diameter near the cardiac orifice were entirely eroded, the peritoneal only remaining, with very few vessels remaining on its surface. The surrounding parts, on the contrary, were deeply vascular. Ulceration had also taken place near the pylorus. The lungs, heart, and œsophagus were healthy. The right kidney was sacculated, the tubular portion being entirely absent; the left was larger than usual. No symptoms of renal disease had been noticed in her lifetime. The brain was not examined, intelligence having remained perfect to the last.

The chief points of interest in this case are: the quantity of chloroform taken, without causing speedy death; the long time (about twelve hours) of total insensibility; the permanence of odor in the breath; and the entire absence of head symptoms on the recovery from the quasi-intoxication. It is not improbable but that she would have recovered, had the chloroform not been taken on an empty stomach, undefended from its erosive action.

The suggestion here forces itself upon one's mind—Might not the anodyne properties of chloroform be safely elicited in the old way of administering remedies, by the mouth—say in the form of chloric ether, in small quantities, mixed in mucilage, every quarter of an hour, till the anæsthetic action is produced?

